# Orienting dilute thin films of non-planar spin-1/2 vanadyl–phthalocyanine complexes†

**DOI:** 10.1039/d2ma00157h

**Published:** 2022-05-14

**Authors:** Zhewen Xu, Vladyslav Romankov, Andrin Doll, Jan Dreiser

**Affiliations:** Swiss Light Source, Paul Scherrer Institut Forschungsstrasse 111 CH-5232 Villigen PSI Switzerland jan.dreiser@psi.ch

## Abstract

The molecular orientation as well as the electronic and magnetic properties of vanadyl–phthalocyanine (VOPc) diluted into titanyl–phthalocyanine (TiOPc) thin films on Si(100) and polycrystalline aluminum substrates have been investigated by soft X-ray absorption spectroscopy (XAS), X-ray linear dichroism (XLD) and X-ray magnetic circular dichroism (XMCD). On the bare substrates the films grow with a standing-up geometry. By contrast, on template layers of 3,4,9,10-perylene-tetracarboxylic dianhydride (PTCDA), they assume a lying-down orientation. Moreover, a theoretical model based on the normalized intensity of the nitrogen K-edge XLD is established in order to extract the molecular orientation angle quantitatively without the need for crystallinity and with the sub-monolayer sensitivity of soft-XAS. XMCD reveals that the vanadium magnetic properties are preserved in both non-diluted and diluted films. The results pave the way toward the use of VOPc as nanometer-sized spin quantum bits.

## Abstract 2

We have found that in dilute films of VOPc in a TiOPc host the molecular orientation can be tailored using a PTCDA layer. An analytical model allows to extract the molecular orientation from the angle-dependent X-ray linear dichroism.

## Graphical abstract

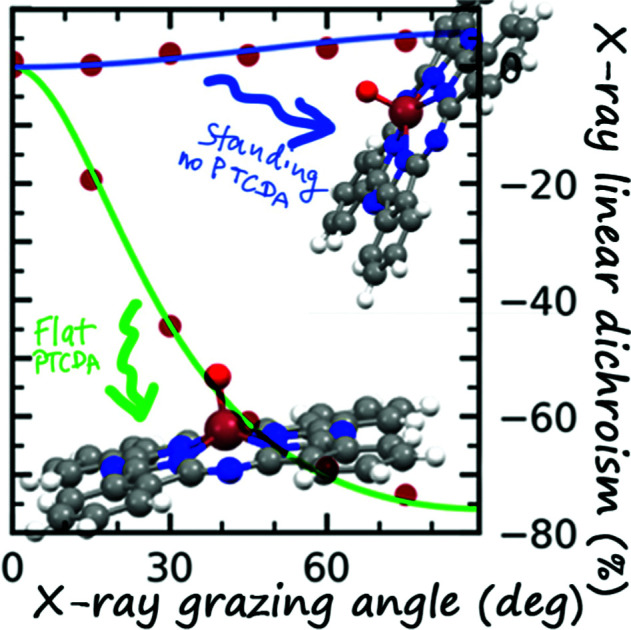

## Introduction

At present there is enormous interest in quantum technologies which are anticipated to deliver unprecedented computational power as well as ultrahigh sensitivity in certain measurements, which is impossible to achieve in classical schemes. While many realizations of quantum bits (‘qubits’) are rather bulky and thus space consuming which is problematic for the integration of large qubit densities, molecular magnets allow for the ultimate miniaturization because of their nanometric size.^1–4^ One way to realize such molecular quantum bits is the use of transition metal or lanthanide complexes. To this end, Rabi oscillations and long-lived quantum coherence of the nuclear spin and/or the electronic spin in the metal center ions have been demonstrated in dilute copper–phthalocyanine (CuPc) molecular films^5^ and in dilute vanadyl–phthalocyanine (VOPc) polycrystalline powder^6^ as well as in other lanthanide containing complexes.^7,8^ Dilution of the spin-carrying molecules in the isostructural diamagnetic host is of high importance here in order to increase the separation and reduce the interaction between the spins promoting larger spin coherence times. The VOPc molecules are particularly interesting in this regard as quantum coherence at room temperature was observed^6^ and coherent coupling to a microwave resonator was demonstrated recently.^9^

Beyond the strong potential for quantum technologies the non-planar VOPc and titanyl–phthalocyanine (TiOPc) alike are raising a lot of interest because of promising applications in organic electronics,^10–12^ gas sensors^13^ and solar cells.^14,15^ For most applications it is desired to control the orientation of the molecules in a film. In monolayer and few-layer heterostructures non-planar phthalocyanine molecules are most often oriented flat with their macrocycles parallel to the substrate plane.^16–20^ Upon increasing the thickness and on non-metallic substrates the molecules tend to assume a ‘standing-up’ geometry.^21–23^ In order to grow films with a flatter orientation of the molecular macrocycles it has been shown that suitable templating layers can be applied for this purpose.^24–26^ Among others, 3,4,9,10-perylenetetracarboxylic dianhydride (PTCDA)^27,28^ is known to function as a templating layer for transition metal and lanthanide phthalocyanine complexes.^29–35^

Synchrotron radiation based polarization dependent X-ray absorption spectroscopy (XAS) or near edge X-ray absorption fine structure (NEXAFS) has been widely used in the past decade in order to investigate the electronic and magnetic structure of monolayers and thin films of metal phthalocyanines on various substrates. Its strengths are the elemental selectivity, which allows the properties of each element to be addressed separately because of the strong resonances occurring in the soft X-ray range which comprises photon energies of about 200 eV up to 2 keV. Among others, this energy range includes the nitrogen K-edge as well as the transition metal L_2,3_ edges. If linearly polarized X-rays with varying polarization direction are employed (X-ray linear dichroism, XLD),^36^ the measurement is mainly sensitive to the filling and orientation of the molecular orbitals. Specifically, at the nitrogen K-edge this reveals the direction along which the π*-orbitals of the phthalocyanine macrocycles can be excited providing a direct link to the orientation of the macrocycles with respect to the X-ray beam. Nitrogen K-edge XLD has been used in the past to study the orientation of thin films of some flat metal-phthalocyanines on different substrates including PTCDA.^30,33,35^ Furthermore, using circularly polarized X-rays, the element specific magnetic properties can be investigated (X-ray magnetic circular dichroism, XMCD).^36^ Another feature of XAS when using the total electron yield mode is the ultrahigh sensitivity due to the very high absorption cross sections in the soft X-ray range, which allows extremely low molecular coverages down to sub-monolayers to be studied.

In the present work we study the effect of PTCDA templating layers on the growth of non-diluted and diluted VOPc and TiOPc films of different thicknesses and compositions. We demonstrate that the templating effect is equally obtained for non-diluted films as well as for VOPc molecules diluted into TiOPc films, and that the magnetic properties of VOPc are preserved. Furthermore we establish a theoretical model that relates the molecular tilt angle with the normalized intensity of the N K-edge XLD quantitatively. Using simple assumptions this model allows the molecular angle in phthalocyanine thin films to be determined in a straightforward manner without the need for crystallinity and with sub-monolayer sensitivity, and it could be generalized to other related materials systems.

## Results and discussion

### Sample overview

Two sets of samples including VOPc and TiOPc were investigated: the first set was grown on Si(100) substrates while for the second one polycrystalline aluminum foil was used, since this substrate material is of relevance for a sensitive spin readout.^37^ The layer sequences were otherwise identical. The detailed sample compositions are given in Table 1. The sample code pattern is ‘MMTTT[-P]-SS’ where ‘MM’ refers to the molecular material (VOPc or TiOPc), ‘TTT’ contains the thickness of the Pc film in nanometers, ‘P’ indicates the presence of a PTCDA templating layer and 'SS' denotes the substrate. The given thicknesses are the nominal thicknesses obtained from quartz crystal microbalance measurements during the sample growth. In part of the samples and in CuPc test samples the thickness was additionally investigated using a profilometer confirming the nominal thickness within an experimental uncertainty of ±16% (*cf.* ESI†).

**Table 1:** Sample composition. The nominal thickness refers to the VOPc/TiOPc layer

Sample name	VOPc [%]	TiOPc [%]	PTCDA [nm]	Substrate	Nominal thick. [nm]
V10-Si/Al	100	0	0	Si(100)/Al	10
V10-P-Si/Al	100	0	10	Si(100)/Al	10
T10-Si/Al	0	100	0	Si(100)/Al	10
T10-P-Si/Al	0	100	10	Si(100)/Al	10
VT10-P-Si/Al	10	90	10	Si(100)/Al	10
VT100-P-Si/Al	10	90	10	Si(100)/Al	100
VT500-P-Si/Al	10	90	10	Si(100)/Al	500

### Nitrogen K-edge spectroscopy

**Fig. 1:**
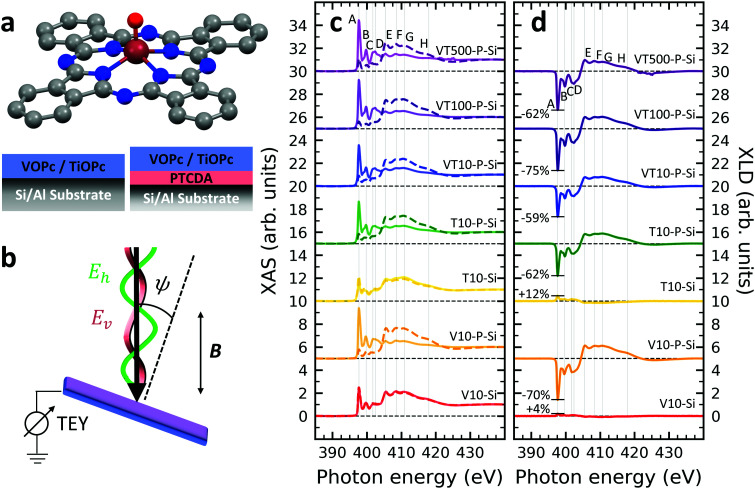
(a) Ball-and-stick model of the metal(iv)-oxo-phthalocyanine molecules. Color code: brown: V or Ti; blue: N; red: O; grey: C. H atoms have been omitted for clarity. Below, a schematic illustrates the two types of grown layer sequences. (b) Measurement geometry. X-rays are incident with grazing angle *ψ* with horizontal or vertical linear polarization. The *B* field is applied collinear with the X-ray beam. (c) XAS and (d) XLD at the nitrogen K-edge recorded on the samples grown on Si substrates (*cf.*Table 1). Spectra were measured at a grazing angle *ψ* = 60° and at room temperature, except for VT500-P-Si (*T* = 2 K). In (c and d) solid (dashed) lines indicate that horizontal (vertical) X-ray polarization was employed for the X-ray spectra. The spectra in (c) and (d) have been vertically offset for clarity. In (d) the XLD intensities normalized to the corresponding XAS are given in percent.

A structural scheme of the metal oxo-phthalocyanine macrocycle is depicted in Fig. 1a revealing the non-flat geometry due to the oxygen atom pointing out of the molecular plane. In addition the measurement geometry is shown in Fig. 1b. The electric field of the X-rays is in the sample plane in the case of linear vertical polarization, and it is partially out-of-plane for linear horizontal polarization and non-zero grazing angles *ψ*. Fig. 1c and d depicts the grazing-incidence nitrogen K-edge XAS and XLD of the samples described in Table 1 grown on Si(100). The linear horizontal (LH, solid lines) and linear vertical (LV, dashed lines) polarizations of the X-rays probe the excitations with the dipole moment pointing out of the sample plane and in the plane, respectively. The XLD is defined as the difference between the LV and LH polarized absorption spectra, that is, XLD = *μ*_v_ − *μ*_h_.

An inspection of the nitrogen K-edge spectra (*cf.*Fig. 1c and d) reveals that the VOPc and TiOPc films without templating layer, *i.e.*, samples V10-Si and T10-Si, show hardly any XLD contrast neither at the π* resonances (398–405 eV) nor at the σ* resonances (above 405 eV).^38^ By contrast, when a templating layer of PTCDA is inserted between the substrate and the non-planar phthalocyanines as in samples V10-P-Si and T10-P-Si, a significant XLD emerges. The characteristic peaks are marked with capital letters A to H. On the one hand, the LH polarized spectra *μ*_h_ exhibit the prominent peaks labelled A to D in Fig. 1c and d that are ascribed to the transitions from the nitrogen 1s to the π* molecular orbitals.^39–41^ On the other hand, *μ*_v_ exhibits the characteristic peaks E to H that originate from the transitions to the σ* molecular orbitals.^39–41^ The excitations are directional because both VOPc and TiOPc contain a sp^2^-hybridized macrocycle, where the π electron cloud extends perpendicularly to the molecular plane and the σ electron cloud is spread in that molecular plane. As a result, for molecules having their macrocycle plane parallel to the one of the substrate at grazing incidence of the X-rays the π* and σ* states can be predominantly excited with LH and LV X-ray polarizations, respectively.^38^

Reversing this argument, negative XLD signals at the π* resonances and positive XLD signals at σ* resonances indicate a flat, lying-down geometry of the molecules. An XLD contrast with inverted signs at π* and σ* resonances indicates a ‘standing-up’ geometry. The appearance of XLD in both π* and σ* resonances after sandwiching the template layer indicates that PTCDA alters the orientation of both VOPc and TiOPc from a ‘standing-up’ configuration to a more ‘lying-down’ configuration. Later on in this paper, a quantitative comparison of the lying-down and the standing-up configurations will be established using a theoretical model. Note that adding a templating layer leads to a significant XLD also in the diluted samples VT10-P-Si, VT100-P-Si, VT500-P-Si. The characteristic peaks of the diluted samples at both π* resonances and σ* resonances resemble the ones of the non-diluted counterparts.

The main features in all spectra shown in Fig. 1c and d are the same, however, very small peak shifts and intensity differences can be ascribed to the slightly different local environments seen by the nitrogen atoms between VOPc and TiOPc.

Furthermore, when using polycrystalline aluminum foil as a substrate (sample identifiers ending with ‘…-Al’), the results are similar, but not identical to the ones obtained with Si(100). The differences will be discussed more quantitatively at the end of the Theoretical modeling section. The respective XAS and XLD spectra are reported in Fig. S1 in the ESI.†

### XAS at the vanadium L_2,3_-edges and the oxygen K-edge

**Fig. 2:**
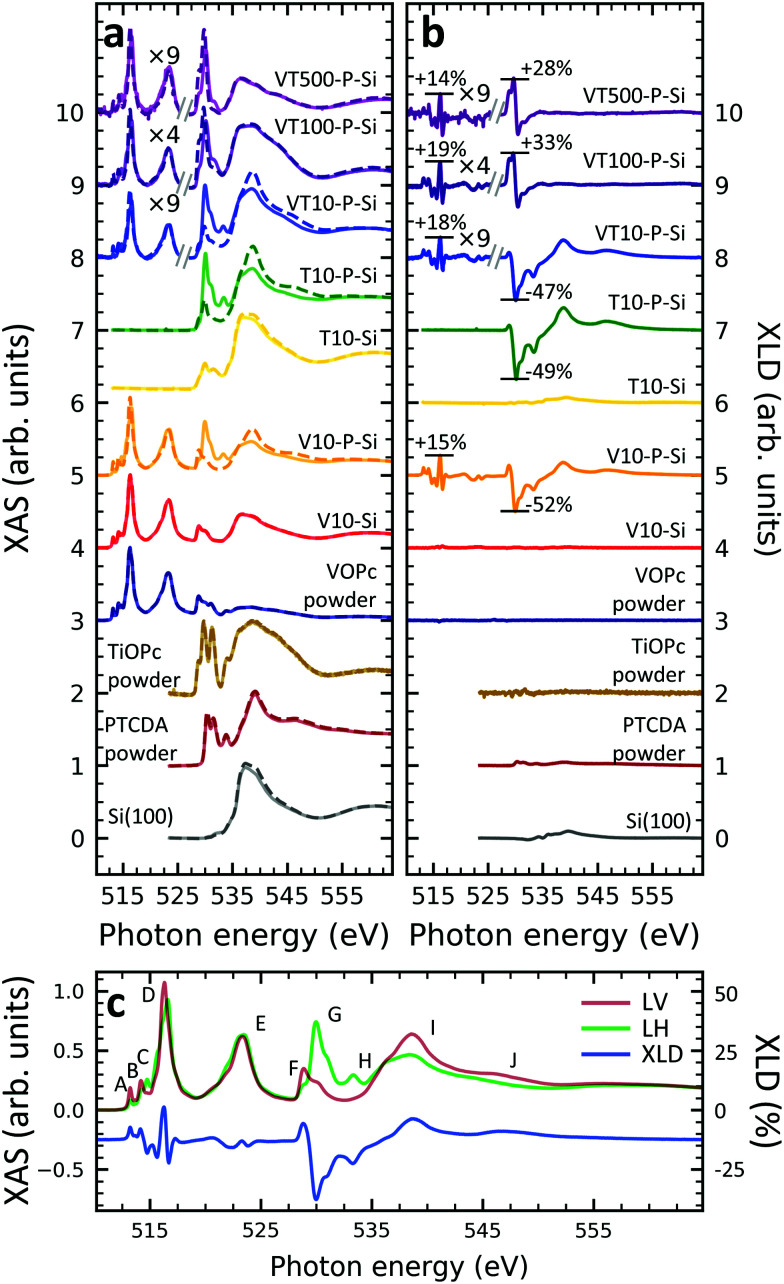
(a) XAS and (b) XLD at the vanadium L_2,3_-edges and at the oxygen K-edge for the indicated samples. Spectra were measured at a grazing angle of *ψ* = 60° at room temperature, except for sample VT500-P-Si (2 K, *ψ* = 60°) and the MOPc powders (300 K, *ψ* = 0°). Solid and dashed lines in (a) represent linear horizontal and vertical X-ray polarization. Spectra in (a) and (b) have been vertically offset for clarity. In (b) the XLD intensities at the main features relative to the corresponding XAS are given in percent. (c) Zoom of the XAS and the XLD of sample V10-P-Si. The XLD scale is relative to the maximum feature of the XAS.

In order to gain deeper understanding beyond the spectral properties of the nitrogen atoms in the molecules, the XAS and XLD spectra at the vanadium L_2,3_-edges (510–527 eV) and the oxygen K-edge (527–560 eV) of the vanadium-containing thin film samples on Si(100) and of a few reference samples were studied. The resulting X-ray spectra are shown in Fig. 2a and b, respectively. In order to improve the visibility, wherever indicated, the vanadium part of the spectra obtained on the diluted VOPc films has been multiplied by a given factor to compensate for the weaker signal. Four main groups of features at photon energies of 515 eV, 523 eV, 530 eV and 538 eV are visible: The first two correspond to the vanadium L_3_ and L_2_ edges originating from the spin–orbit split core-level electron transitions 2p_3/2_ → 3d and 2p_1/2_ → 3d, respectively.^36^ The third and the fourth groups belong to the oxygen K-edge, which will be discussed further below. The vanadium L_2,3_-edge spectra of the non-diluted VOPc samples V10-Si and V10-P-Si are in excellent agreement with the ones in the literature.^39,42–44^

In line with the results obtained at the nitrogen K-edge discussed before it is apparent that without a templating layer there is virtually no XLD signal. In contrast, when a PTCDA layer is introduced the characteristic XLD signal appears. Obviously, the appearance of the XLD effect observed at the vanadium and nitrogen edges must be correlated because both elements are engaged in the same molecule and fixed by chemical bonds. The diluted samples exhibit a behavior similar to the non-diluted VOPc sample V10-P-Si. Fig. 2c provides a detailed view of the XAS and XLD features of sample V10-P-Si. Peaks A, B and D (*cf.*Fig. 2c) exhibit a positive XLD signal, while the XLD at peak C is negative.

According to the literature the main peak D is assigned to excitations into the 3d_*x*^2^−*y*^2^_ orbital of the V^4+^ ion.^39^ Peak C can be ascribed to the transition into the vanadium 3d_*z*^2^_ orbital,^39^ which is oriented perpendicular to the plane of the molecular macrocycle. This corroborates the above conclusion drawn from the nitrogen K-edge XLD that the VOPc thin films prefer to lie down on PTCDA/Si. Similar results are also found for the VOPc containing films grown on PTCDA/Al (*cf.* Fig. S2 of the ESI†). As already mentioned the feature labeled with 'E' corresponds to the vanadium L_2_ edge containing little information in addition to the L_3_ edge.

A comparison with the powder samples and the thick films on PTCDA as shown in Fig. 2a yields that peaks H, I and J are predominantly related to the oxygen excitations in the PTCDA layer. Features F and G contain overlapping signatures from both PTCDA and VOPc. Part of the signal at peaks F and G can be assigned to the anti-bonding π* and σ*, respectively, orbitals of the oxygen atom bound to the vanadium ion. The VOPc/TiOPc contribution becomes clearly visible only in the 100 nm and 500 nm diluted films (*cf.*Fig. 2a and b).

While keeping in mind the overlap of phthalocyanine and PTCDA features, some orientational information can also be deduced from the O K-edge. In both VOPc and TiOPc, oxygen forms a double bond with the transition metal, with the bond axis perpendicular to the molecular plane of the phthalocyanine. Therefore, the corresponding π* (below ≲530 eV) and σ* (above ≳530 eV)^39^ resonances are excited predominantly with the X-ray electric field in the macrocycle plane and out-of-plane, respectively, opposite to the nitrogen case. Correspondingly, the behavior in terms of the sign change of the oxygen K-edge XLD is reversed with respect to the one at the nitrogen K-edge for a given molecular orientation. Without the PTCDA template, the non-diluted VOPc and TiOPc samples V10-Si and T10-Si show almost no XLD consistent with what is observed for nitrogen and vanadium. Also in the case of the oxygen K-edge, with PTCDA a strong XLD appears. Precisely, at peak F the XLD is positive, whereas it is negative at peak G. This again confirms that VOPc and TiOPc favor a ‘lying-down’ configuration on PTCDA/Si and PTCDA/Al regardless of film thickness and dilution.

An inspection of the thickness dependence yields that in the 10 nm-thick films with a PTCDA layer (V10-P-Si, T10-P-Si, VT10-P-Si) the oxygen K-edge features of PTCDA (*cf.*Fig. 2c, peaks H, I, J) are observed. In contrast to the powder reference, which does not exhibit any XLD because of the random orientation of the molecules, an XLD appears in the PTCDA signature of the thin film samples as expected owing to the orientation of the PTCDA molecules on the substrate. Upon increasing the thickness from 10 nm to 100 nm (sample VT100-P-Si) and further to 500 nm (VT500-P-Si) the characteristic XLD features of PTCDA disappear (*cf.*Fig. 2b). The PTCDA related XLD features appear in the non-diluted and diluted 10 nm-thick films despite the lower probing depth in the total electron yield mode of ∼5 nm.^36^ This can be explained by a spatially inhomogeneous growth of VOPc and TiOPc on the templating layer. Indeed, even at a thickness of tens of nanometers films appear still rather grainy as observed by atomic force microscopy studies on VOPc films^45,46^ and on few-nm thin CuPc films.^47^ Similarly, in the 10 nm thick films grown without a templating layer (V10-Si and T10-Si) signatures of the oxygen K-edge of the surface-oxidized Si(100) substrate are observed at a photon energy of 536.7 eV. Consistently, with increasing amount of deposited molecules, the characteristic features belonging to the oxygen K-edge of PTCDA disappears.

### XAS at the titanium L_2,3_-edges

**Fig. 3:**
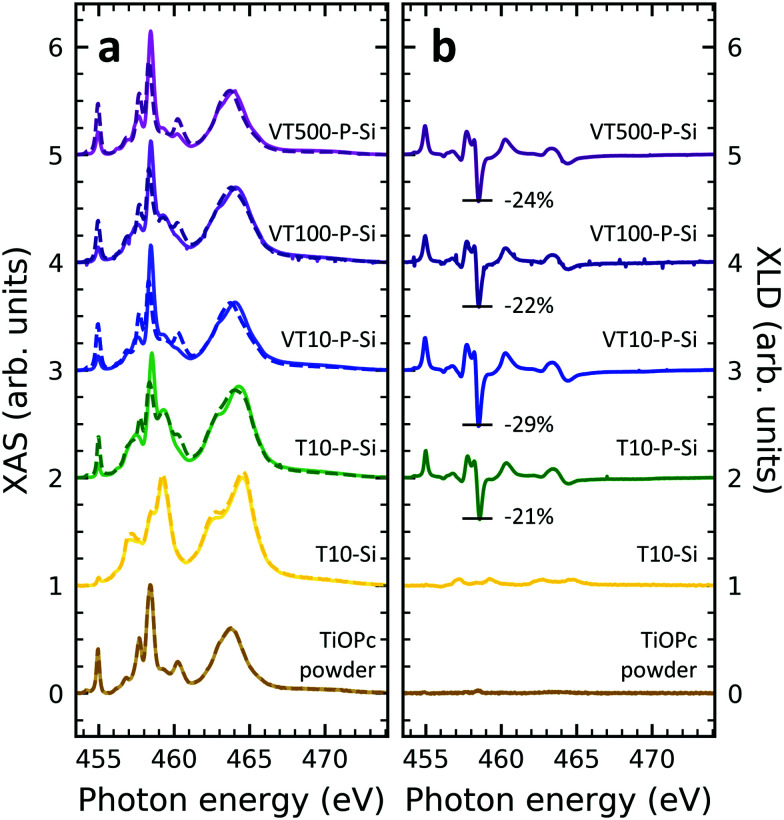
(a) XAS and (b) XLD at the titanium L_2,3_-edges of thin film and reference samples as indicated in the plot. Spectra were recorded at grazing angle *ψ* = 60° and at room temperature except for the diluted samples, which were measured at 2 K. In (a) XAS, solid/dashed lines represent linear horizontal/vertical polarization of the X-rays. Spectra have been vertically offset for clarity. In (b) the XLD intensities normalized to the corresponding XAS are given in percent.

In order to obtain insight into the properties of Ti-containing samples, XAS at the Ti edges were recorded. Fig. 3 illustrates the XAS and XLD at the titanium L_2,3_-edges of different titanium-containing thin films and reference samples as indicated. Analogous results obtained on the aluminum foil substrates are shown in Fig. S3 (ESI†). The spectral features in Fig. 3a can be divided into two groups: the group of peaks at photon energies below and above ∼460 eV ^48^ are attributed to the L_3_ and L_2_ edges, respectively. A clear separation is difficult because of the rather low spin–orbit coupling determining this peak separation. Similar to the vanadium spectra, within each group the strength of the crystal field imposed by the surrounding nitrogen and oxygen atoms and its symmetry determines the splitting into further peaks.^49,50^ Evidently, without templating layer the non-diluted TiOPc thin film on Si barely shows any XLD contrast as visible in Fig. 3b. With PTCDA a strong XLD emerges also at the titanium L-edges. In addition, the titanium XAS of TiOPc powder resembles the spectrum in literature.^51^ Also, the XAS of the TiOPc thin films are consistent with the ones in the literature confirming the high quality of the samples.^50,51^

Focusing on the diluted samples on both substrates of Si and Al, it is obvious that both XAS and XLD spectra exhibit well-resolved peaks at both titanium L_2,3_-edges, which are quite similar to the ones of non-diluted TiOPc with PTCDA. Furthermore, these XAS (Fig. 3a and Fig. S3a, ESI†) and XLD (Fig. 3b and Fig. S3b, ESI†) spectra of diluted samples are almost identical in shape and magnitude, regardless of substrates and sample thickness. It clearly demonstrates that these films of diluted paramagnetic molecules in the diamagnetic matrix are of high quality with a thickness independent mass ratio of the two molecular species.

### Theoretical modeling: molecular angle from nitrogen XLD

Given the high structural complexity that can occur in phthalocyanine films^46^ it is highly desirable to derive simple, yet important structural parameters from the present XLD data. Certainly, detailed information can be obtained from X-ray diffraction (XRD) measurements, however, these are not possible or at least extremely time-consuming for few-nm thin films. Furthermore, XAS/XLD has ultrahigh sensitivity down to the sub-monolayer range, and it does not require any crystallinity in contrast to XRD. In the following we will show that the molecular angle *θ* (see below) of an azimuthally disordered film of phthalocyanine molecules can be quantitatively derived from the strength of the XLD signal at the nitrogen K-edge using straightforward and simple assumptions. The grazing-angle dependent XLD obtained on samples V10-Si and V10-P-Si will serve to validate the model. Specifically, the model relates the angle *θ* to the intensity of the main π* transition of the XLD spectra at a photon energy of 397.7 eV. Because of its generality, this model can be applied to any other metal phthalocyanine thin film, regardless of their planar or non-planar nature.

**Fig. 4:**
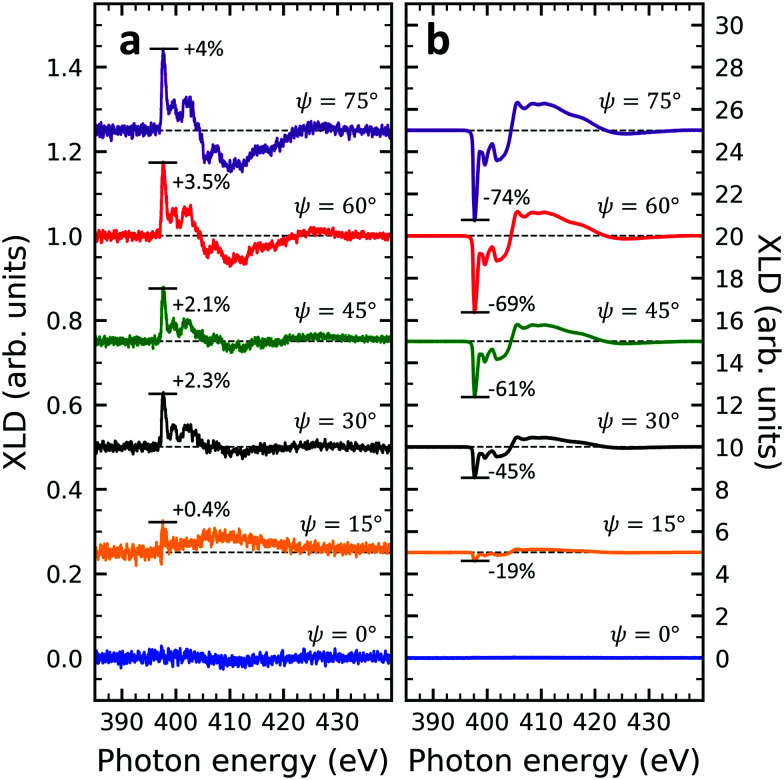
Nitrogen K-edge XLD of samples (a) V10-Si and (b) V10-P-Si at different grazing angles ranging from *ψ* = 0° to 75° in 15° steps. Spectra have been vertically offset for clarity. The XLD intensities normalized to the corresponding XAS are given in percent. The spectra in (a) appear to be more noisy than in (b) because of the big difference in the vertical scale.

XLD spectra at the nitrogen K-edge of samples V10-Si and V10-P-Si are depicted as a function of grazing angle *ψ* in Fig. 4. For both samples a very clear *ψ* dependence is observed. Upon increasing *ψ*, the XLD signals become more and more distinct. Obviously, the two samples exhibit a similar shape but the opposite sign of the XLD. As it has been discussed before, the negative (positive) XLD π* signal peaking at a photon energy of 397.7 eV indicates a ‘lying-down’ (‘standing-up’) geometry of the molecular macrocycles on the substrate.^38^

**Fig. 5:**
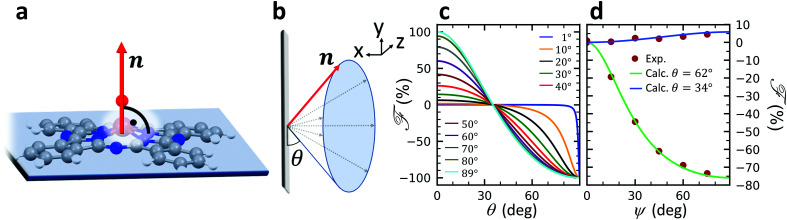
(a) Geometry used in the model. **n** denotes the unity vector normal to the molecular plane. (b) The molecules are assumed to be oriented with a random azimuthal angle on the substrate plane drawn in grey. The molecules' normal vectors **n** form an angle *θ* with the substrate plane. Hence *θ* = 0° corresponds to the molecular standing-up geometry and *θ* = 90° corresponds to the lying-down orientation. (c) Calculated figure of merit ℱ as a function of angle *θ* at different grazing angles *ψ* as indicated in the plot. (d) Symbols and solid lines (color code: V10-P-Si, green, *θ* = 62° and V10-Si, blue, *θ* = 34°) indicate the measured and calculated XLD figure of merit ℱ showing excellent agreement.

The full derivation of the model is described in detail in the Supplementary Information. The geometry is illustrated in Fig. 5a and b: due to azimuthal disorder of molecular crystallites, the molecular normal vectors **n** form a cone with an angle *θ* to the substrate plane. Here, we mention only the final expression which relates the molecular angle *θ*, the X-ray grazing angle *ψ* as shown in Fig. 1 and the XLD figure of merit ℱ which will be explained just below:1
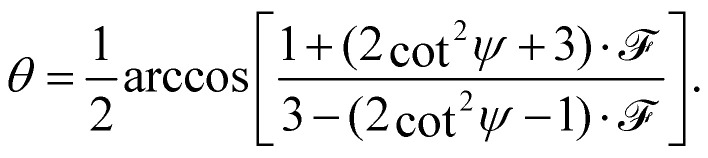
Here, ℱ is a quantity characterizing the normalized intensity of the XLD at the π* resonance maximum. It is defined as 
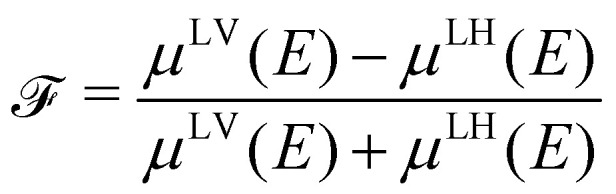
 at *E* = 397.7 eV. Fig. 5c illustrates the calculated behavior of ℱ as a function of the angle *θ* for different X-ray grazing angles obtained using eqn (1). Note that all curves form a nodal point at *θ*_node_ = arccos(1/3)/2 = 35.3° which indicates that at *θ*_node_ the figure of merit value becomes independent of the grazing angle *ψ* and is always identical to ℱ = 0. Furthermore, it is observed that at small grazing angles *ψ* the sensitivity of determining *θ* from ℱ is very low.

In order to prove the validity of the model eqn (1) the molecular angle *θ* was determined for both VOPc 10 nm-thin films with and without templating layer, V10-Si and V10-P-Si, given the measured XLD figure of merit values ℱ(*ψ*). To this end, for each experimental value of ℱ for all given non-zero grazing angles *ψ* the molecular angle *θ* was calculated using eqn (1). The results are depicted in Fig. 5d. Taking the average and standard deviation of the *θ* values yields an angle *θ* for the sample V10-P-Si with the PTCDA templating layer of *θ*_V10-P-Si_ = 62.4° ± 0.8°. On the other hand, without templating layer a smaller molecular angle *θ*_V10-Si_ = 33.7° ± 0.6° is obtained. The low uncertainties demonstrate the good agreement of the model with the experimental data. This is further corroborated by the calculated *ψ*-dependencies for the two samples shown as solid lines in Fig. 5d. Note that the experimental error in obtaining the molecular angles is rather dominated by the mounting accuracy of the samples in the measurement chamber, which is estimated to be on the order of ±1°. Furthermore, the excellent match of the calculated curves for the two best-match values for *θ* corroborates this result and provides confiance in the modeling and in the initial assumptions of a homogenous tilt angle with azimuthal disorder of molecular crystallites.

The *θ*_V10-P-Si_ value found from the calculation is in excellent agreement with the literature result, where an angle of 62° was found, *i.e.*, 28° measured from the substrate normal.^46^ The obtained molecular angles of the above two samples demonstrates in accordance with reference^46^ that the template layer PTCDA can significantly change the orientation of VOPc from a ‘standing-up’ orientation to a more lying-down orientation. Note that the molecular angles obtained are the ones of the topmost molecular layers because of the surface sensitivity of the used XAS technique in total electron yield mode with a probing depth of ∼5 nm.^36^ Furthermore, it is interesting to note that the excellent agreement of the *θ*_V10-P-Si_ value with the literature result occurs while there could be other crystalline phases present. From our results we conclude that the model works well as long as there is a dominant contribution of a certain phase. We emphasize that the described model offers a very simple yet powerful and efficient approach that could be generalized toward other metal phthalocyanines on different substrates.

Using the model described in the above eqn (1), the molecular angles in the VOPc and TiOPc thin films as a function of different influencing factors such as substrate type, dilution as well as the film thickness are quantitatively obtained. Table 2 provides the experimentally obtained molecular angles *θ* for the different studied samples.

**Table 2:** Experimental XLD figure of merit values ℱ and molecular angles *θ* of the different samples studied in this work. The experimental error in determining the molecular angle values is estimated to ±1.5°

Sample	ℱ [%] on Si	*θ* on Si	ℱ [%] on Al	*θ* on Al
V10-Si/Al	4.0	34°	18.7	28°
V10-P-Si/Al	−69.5	62°	−57.9	57°
T10-Si/Al	12.3	31°	16.8	29°
T10-P-Si/Al	−62.3	59°	−45.9	52°
VT10-P-Si/Al	−59.4	57°	−49.5	53°
VT100-P-Si/Al	−74.6	65°	−62.9	59°
VT500-P-Si/Al	−61.6	58°	−60.8	58°

It is evident that without the PTCDA template layer the molecules in the 10 nm-thin films form an angle in the range of 28°–34° depending on the type of substrate. When PTCDA is sandwiched between the substrate and the molecular thin films, the molecular angles are increased dramatically by about 30°. It is also worth noting that, first, the films deposited on the Si substrates exhibit a few degrees larger molecular angle compared to the ones grown on aluminum foil. Second, it appears that in most cases of the 10 nm-thin films the VOPc molecules are slightly more ‘lying-down’ oriented than the TiOPc ones independent of the substrate or the presence of PTCDA. Third, in the diluted thin films the film thicknesses do not have a strong influence. Upon increasing the thickness a slight variation of the molecular angle is observed. Although this trend is similar for the films grown on aluminum foil, the differences are even smaller in that case, falling below the error bars.

**Fig. 6:**
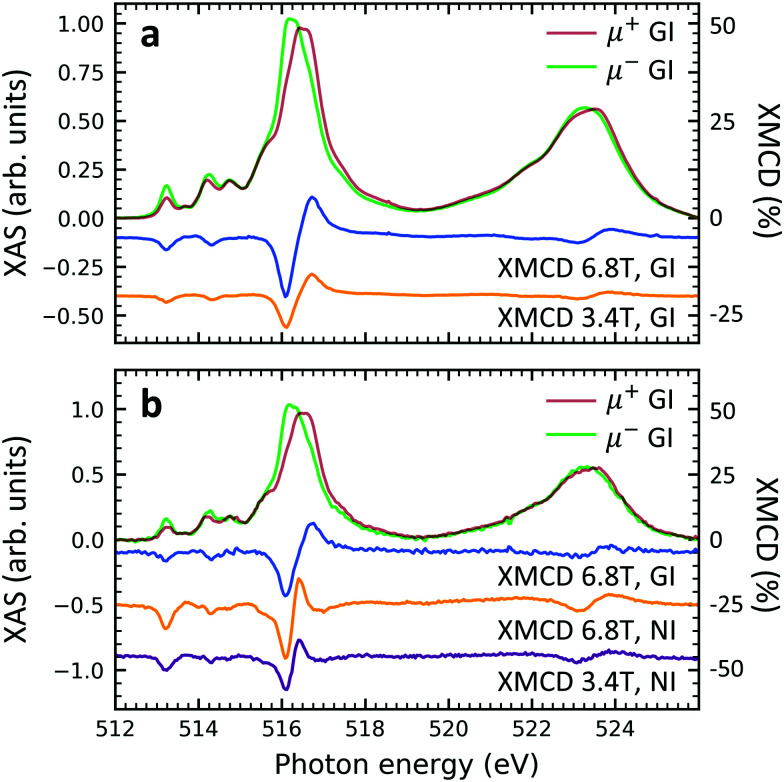
Circularly polarized XAS and XMCD at the vanadium L_2,3_-edges of (a) sample V10-P-Si and (b) sample VT100-P-Si. Measurements were performed at 2 K. The magnetic fields and incidence angles of *ψ* = 60° (GI, grazing) or *ψ* = 0° (NI, normal) are indicated in the plots. The XMCD curves have been vertically offset to improve visibility. The XMCD scales are relative to the maximum features of the XAS.

### X-ray magnetic circular dichroism

Element specific XMCD measurements were performed at low temperature (2 K) and high magnetic field (6.8 T) in order to investigate the thin films' magnetic properties (Fig. 6). The shape and the strength of the characteristic XMCD signals at the vanadium L_2,3_-edges^44,52–54^ are equally observed in both, non-diluted and diluted, samples. This result suggests that the spin *S* = 1/2 of the vanadium 3d shell is well preserved. Furthermore, the insertion of the PTCDA layer does not lead to the loss of the isolated spin 1/2 in the VOPc molecules (*cf.* Fig. S6 of the ESI†). When comparing circularly polarized XAS and XMCD spectra of individual samples measured at different strengths of external magnetic fields, *i.e.*, 3.4 T and 6.8 T, it is found that the magnitudes of the circularly polarized spectra at the vanadium L_2,3_ edges stay more or less unchanged, while their difference (XMCD) almost doubles. This indicates an increase of the vanadium magnetic moment by a factor of close to two, which is compatible with a spin 1/2 and a temperature of 2–3 K in accordance with SQUID measurements.^39^ The fact that upon doubling the applied field the magnetic moment increases by a factor of slightly less than two indicates the onset of saturation as expected for a paramagnetic spin *S*= 1/2. Note that due to the spectral overlap between the vanadium L_3_ and L_2_-edges as well as the overlap between the vanadium L_2_ and the oxygen K-edges, the applicability of sum rules^55,56^ is limited. A comparison between the grazing and normal incidence XMCD spectra yields that no significant difference appears. Hence, the thin films behave magnetically close to isotropic.

## Conclusion

The electronic structure and magnetism of thin films of VOPc, TiOPc on Si(100) and polycrystalline aluminum substrates with and without a PTCDA interlayer have been investigated. A simple, yet powerful theoretical model is established relating the strength of the π* resonances at the nitrogen K-edge XLD to the orientation angles of the molecular macrocycles in each sample. The introduction of a PTCDA templating layer significantly changes the molecular orientation toward a more ‘lying-down’ geometry where the molecular macrocycles are parallel to the substrate plane. This effect is confirmed by the behavior of the π* and σ* resonances of the nitrogen and oxygen K-edges. The templating effect of PTCDA is also present in 10% diluted thin films of VOPc in TiOPc as witnessed by the XLD signatures. The XMCD measurements reveal that the vanadium ions in VOPc keep their magnetic properties in both non-diluted and diluted samples. This indicates that the spin 1/2 residing in VOPc is robust against external perturbations such as sample thickness, substrate, template layer and dilution. The present results open an avenue toward the use of VOPc molecules as quantum bits in future information processing devices.

## Experimental

### Sample preparation

Substrates of p-type (boron doped) Si(100) (10–30 Ω cm) and commercial polycrystalline aluminum foil with a thickness of 18 μm were subsequently cleaned in acetone and then ethanol in an ultrasonic bath. Afterwards, they were mounted on an aluminum holder and transferred into the deposition chamber (base pressure ∼ 1 × 10^−8^ mbar). Commercial VOPc (Sigma-Aldrich), TiOPc (Tokyo chemical industry Co. Ltd) and PTCDA (Sigma-Aldrich) were loaded into evaporator crucibles and thoroughly degassed. Molecular films were deposited onto the substrates held at room temperature from a Knudsen cell thermal evaporator. The deposition rates were controlled by varying the temperature of the individual crucibles. The thin film thicknesses were estimated by an *in situ* quartz crystal microbalance with ∼6 MHz resonance frequency. The vast majority of the non-diluted samples were grown at deposition rates of about 10 Hz min^−1^. This rate was achieved for VOPc at 280 °C using a quartz crucible and for TiOPc at 295 °C using an aluminum oxide crucible. For a few calibration samples the film thicknesses were checked by profilometer measurements with a scanning force of 0.1 mg. Diluted samples (10% VOPc diluted in TiOPc) were acquired by simultaneously subliming VOPc and TiOPc from two Knudsen cells running in parallel. The dilution ratio of 1 : 9, was achieved by adjusting the VOPc deposition rate to 11% of the TiOPc deposition rate and maintained throughout the whole deposition process.

### XAS, XLD and XMCD Measurements

The X-ray experiments were conducted in the total electron yield (TEY) mode at the X-Treme beamline^57^ of the Swiss Light Source at the Paul Scherrer Institute. The X-ray beam spot size was 1.3 × 1 mm^2^ and the photon flux was attenuated to avoid beam damage. For the XMCD measurements the magnetic field was collinear with the incoming X-ray beam. The XAS signal is defined as the sum of the two corresponding polarized X-ray spectra, that is, XAS = (*μ*_v_ + *μ*_h_) or (*μ*^+^ + *μ*^−^). The XLD is defined as the difference between spectra measured with vertical and horizontal polarization, *i.e.*, XLD = *μ*_v_ − *μ*_h_. Similarly, the XMCD is the difference of spectra measured with left and right circular polarization, *i.e.*, XMCD = *μ*^+^ − *μ*^−^.

The normalization of the spectra for the different elements was performed as follows:

#### N K-edge

For each spectrum, a linear background was subtracted in order to obtain a flat and zero pre-edge baseline. Then the spectra were scaled by a constant factor to normalize the post-edge to one.

#### V L_2,3_ edges (XLD)

After subtracting a linear background, the polarization-averaged spectra of the non-diluted samples containing vanadium (sample identifiers starting with ‘V – …’ as well as VOPc powder sample) were normalized to the main feature of the vanadium L_3_-edge. The polarization-averaged spectra of all other samples were normalized to the strongest features of the oxygen K-edge. The polarized spectra were then scaled accordingly.

#### V L_2,3_ (XMCD) and Ti L_2,3_–edges

After subtracting a linear background the polarization-averaged spectra were normalized to the main features of the vanadium L_3_-edge and the titanium L_3_-edge, respectively. The polarized spectra were then scaled accordingly.

## Author contributions

Z. X.: investigation, data curation, formal analysis, visualization and writing (original draft); V. R.: investigation, supervision; A. D.: investigation, supervision, methodology; J. D.: conceptualization, supervision, methodology, funding acquisition, writing (original draft). All authors discussed the results and commented on the manuscript.

## Conflicts of interest

There are no conflicts to declare.

## Supplementary Material

MA-003-D2MA00157H-s001
