# Supplementary material for: Orienting dilute thin films of non-planar spin-1/2 vanadyl–phthalocyanine complexes
Source: Mater Adv. 2022 May 14;3(12):4938–46. doi: 10.1039/d2ma00157h (PMC9207598; doi:10.1039/d2ma00157h)
Supplement: MA-003-D2MA00157H-s001 [file MA-003-D2MA00157H-s001.pdf]

Electronic Supplementary Material (ESI) for Materials Advances.
This journal is © The Royal Society of Chemistry 2022

Supplementary Information

Orienting Dilute Thin Films of Non-Planar Spin-1/2 Vanadyl-Phthalocyanine Complexes

Zhewen Xu, *Vladyslav Romankov*, *Andrin Doll*, *Jan Dreiser**

Swiss Light Source, Paul Scherrer Institut, Forschungsstrasse 111, CH-5232 Villigen PSI, Switzerland

Email Address: jan.dreiser@psi.ch

1 X-ray absorption spectra for thin films grown on aluminum foil

1.1 Nitrogen K-edge spectra

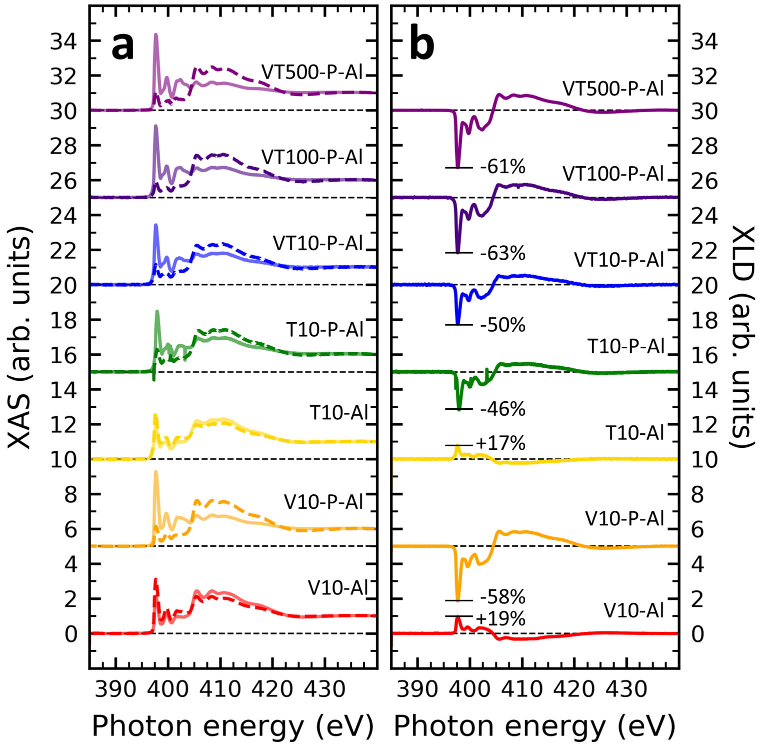

Figure S1. (a) XAS and (b) XLD at the nitrogen K-edge of thin films grown on aluminum foil as indicated in the plot. Measurements were performed at a grazing angle of $\psi = 60^\circ$ and at room temperature, except for sample `VT500-P-Al` which was measured at 2 K. In (a) solid and dashed lines represent linear and vertical polarization of the X-rays, respectively. In (b) the XLD intensities normalized to the corresponding XAS are given in percent.

1.2 Vanadium $L_{2,3}$-edges and oxygen K-edge

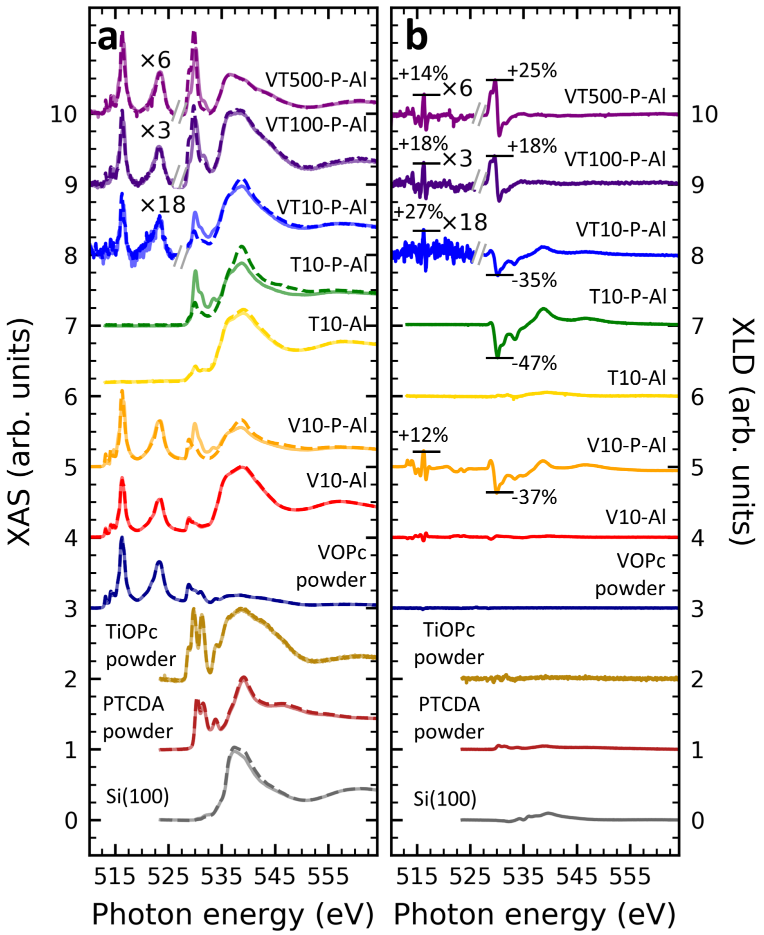

Figure S2. (a) XAS and (b) XLD at the vanadium $L_{2,3}$-edges and at the oxygen K-edge of different thin films grown on aluminum foil and reference samples as indicated in the plot. Spectra were recorded at 300 K at a grazing angle of $\psi = 60°$, except for samples `VT500-P-Al` (2 K, $\psi = 60°$) and the MOPc powders (300 K, $\psi = 0°$). In (a) solid and dashed lines represent linear horizontal and vertical X-ray polarization, respectively. Values in percent given in (b) refer to the XLD intensities of the maximum features normalized to the corresponding XAS.

1.3 Titanium $L_{2,3}$-edges

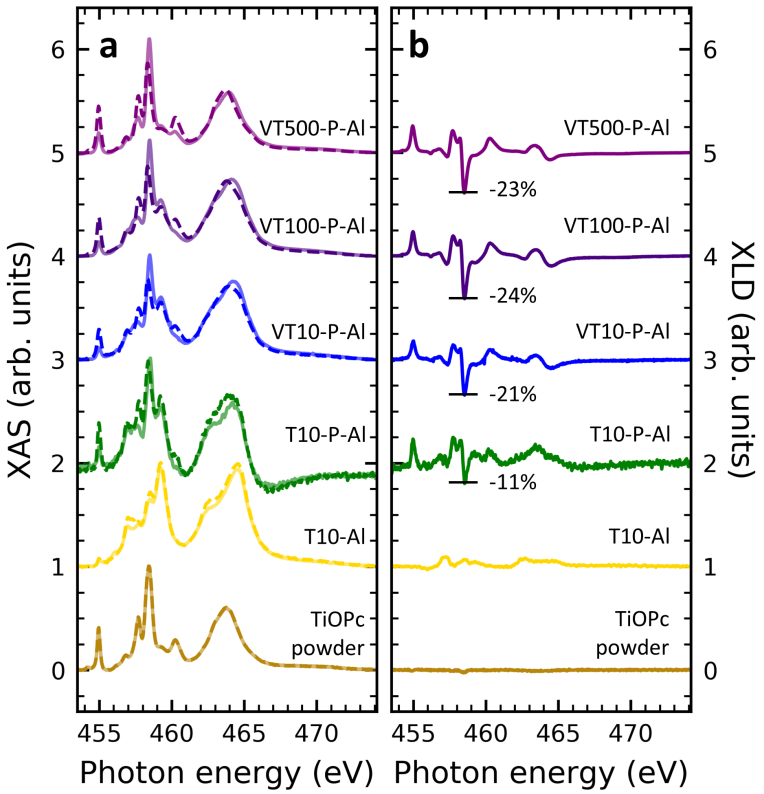

Figure S3. (a) XAS and (b) XLD at the titanium $L_{2,3}$-edges of different thin films grown on aluminum foil and of a TiOPc powder reference. All spectra were recorded at a grazing angle of $\psi = 60°$. The TiOPc powder and the non-diluted TiOPc films were studied at room temperature, whereas the diluted films were measured at 2 K. In (a) solid and dashed lines represent linear horizontal and vertical X-ray polarizations, respectively. Values in percent given in (b) refer to the XLD normalized to the corresponding XAS.

2 Derivation of the theoretical model

In the following derivation the coordinate system shown in **Figure S4** will be used. In spherical coordinates, the molecule's normal vector **n** (**Figure S4**a) reads

$$\mathbf{n} = (\sin\theta, \cos\theta\cos\varphi, \cos\theta\sin\varphi), \tag{1}$$

where θ is the angle between **n** and the substrate plane (polar angle). We assume that the dipole moment of the molecular π^* excitations is oriented along **n**. Furthermore, φ is the corresponding azimuthal angle.

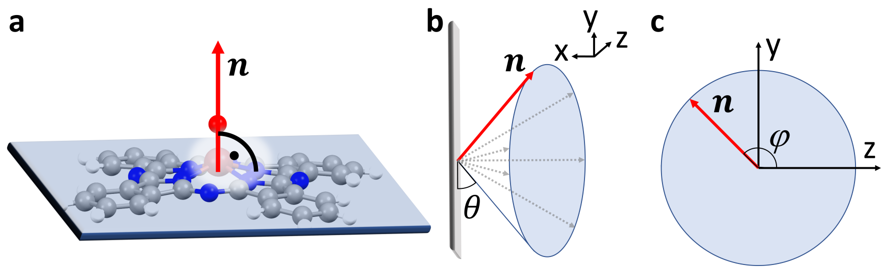

Figure S4. (a) Molecular plane and its normal vector **n**. (b) Substrate plane (grey) and distribution of molecular normal vectors **n** forming a cone with opening angle θ to the substrate plane. (c) Projection along the x axis illustrating the angle φ.

2.1 Case of normal incidence of the X-rays

The normalized vectors of the electric field direction for linear vertical and horizontal X-ray polarizations are written as follows

$$\text{LV}: \quad \mathbf{e_1} = (0, \pm 1, 0), \tag{2}$$

$$\text{LH}: \quad \mathbf{e_2} = (0, 0, \pm 1). \tag{3}$$

The azimuthal averaging of the LV intensity yields

$$\begin{aligned} \mathrm{I}_1 = \frac{1}{2\pi}\int_0^{2\pi} |\mathbf{n}\cdot\mathbf{e}_1|^2\, d\varphi &= \frac{1}{2\pi}\int_0^{2\pi} \cos^2\theta\cos^2\varphi \quad d\varphi \\ &= \frac{1}{2\pi}\cos^2\theta\cdot\int_0^{2\pi}\frac{1+\cos(2\varphi)}{2} \quad d\varphi \\ &= \frac{1}{2\pi}\cos^2\theta\cdot\left.\frac{2\varphi+\sin(2\varphi)}{4}\right|_0^{2\pi} \\ &= \frac{1}{2}\cdot\cos^2\theta. \end{aligned} \tag{4}$$

The azimuthal averaging of the LH intensity yields

$$\begin{aligned} \mathrm{I}_2 = \int_0^{2\pi} |\mathbf{n}\cdot\mathbf{e}_2|^2\, d\varphi &= \frac{1}{2\pi}\int_0^{2\pi} \cos^2\theta\sin^2\varphi \quad d\varphi \\ &= \frac{1}{2\pi}\cos^2\theta\cdot\int_0^{2\pi}\frac{1-\cos(2\varphi)}{2} \quad d\varphi \\ &= \frac{1}{2\pi}\cos^2\theta\cdot\left.\frac{2\varphi-\sin(2\varphi)}{4}\right|_0^{2\pi} \\ &= \frac{1}{2}\cdot\cos^2\theta. \end{aligned} \tag{5}$$

Note that in the case of normal incidence the intensities I_1 and I_2 are identical because the probed sample is symmetric in the yz-plane (azimuthal disorder) and the electric fields of the LV and LH polarized X-rays are both in that plane.

2.2 Case of arbitrary incidence angle ψ of the X-rays

A more general situation is discussed that the incidence of photon is not perpendicular to the substrate any more, but with a particular grazing angle ψ which corresponds to the rotation angle of the substrate (yz-plane) around the y-axis (cf. Figure 1a of the main text). The coordinate system of the substrate and the molecules is kept the same as in the previous case, but the X-ray electric field vector in LH polarization is rotated. Hence the general expression of the molecule's normal vector **n** remains the same as in **Equation 1**. The normalized vector of the electric field in LV polarization is also identical to the previously dicussed **Equation 2**. However, because of the specific measurement geometry, the normalized LH electric field changes its form, where the normal incidence case is recovered in case of $\psi = 0°$:

$$\text{LH}: \quad \mathbf{e_2} = (\sin\psi, 0, \cos\psi), \tag{6}$$

The integral of the LV intensity takes the same expression as Equation 4, on the contrary, the integral of LH intensity becomes

$$\begin{aligned}
\mathrm{I}_2 = \frac{1}{2\pi}\int_0^{2\pi} |\mathbf{n}\cdot\mathbf{e}_2|^2\, d\varphi &= \frac{1}{2\pi}\int_0^{2\pi} |\sin\theta\cdot\sin\psi + \cos\theta\cdot\sin\varphi\cdot\cos\psi|^2\, d\varphi \\
&= \sin^2\theta\sin^2\psi + \frac{1}{\pi}\sin\theta\cos\theta\cdot\sin\psi\cos\psi\cdot\int_0^{2\pi}\sin\varphi\, d\varphi \\
&\quad + \frac{1}{2\pi}\cos^2\theta\cos^2\psi\cdot\int_0^{2\pi}\sin^2\varphi\, d\varphi \\
&= \sin^2\theta\sin^2\psi + 0 + \frac{1}{2\pi}\cos^2\theta\cos^2\psi\cdot\int_0^{2\pi}\frac{1-\cos(2\varphi)}{2}\, d\varphi \\
&= \sin^2\theta\sin^2\psi + \frac{1}{2}\cdot\cos^2\theta\cos^2\psi.
\end{aligned} \tag{7}$$

With the obtained LV intensity (I_1) and LH intensity (I_2), the XLD figure of merit $\mathscr{F}$ is obtained as

$$\begin{aligned}
\mathscr{F} = \frac{\text{LV}-\text{LH}}{\text{LV}+\text{LH}} &= \frac{I_1 - I_2}{I_1 + I_2} \\
&= \frac{(\frac{1}{2}\cdot\cos^2\theta) - (\sin^2\theta\sin^2\psi + \frac{1}{2}\cdot\cos^2\theta\cos^2\psi)}{(\frac{1}{2}\cdot\cos^2\theta) + (\sin^2\theta\sin^2\psi + \frac{1}{2}\cdot\cos^2\theta\cos^2\psi)} \\
&= \frac{-\sin^2\theta\sin^2\psi - \frac{1}{2}\cdot\cos^2\theta(\cos^2\psi - 1)}{\sin^2\theta\sin^2\psi + \frac{1}{2}\cdot\cos^2\theta(\cos^2\psi + 1)} \\
&= \frac{-2\tan^2\theta\sin^2\psi - \cos^2\psi + 1}{2\tan^2\theta\sin^2\psi + \cos^2\psi + 1} \\
&= \frac{-2\tan^2\theta\sin^2\psi + \sin^2\psi}{2\tan^2\theta\sin^2\psi + 2\cos^2\psi + \sin^2\psi} \\
&= \frac{-2\tan^2\theta + 1}{2\tan^2\theta + 2\cot^2\psi + 1}.
\end{aligned} \tag{8}$$

For arbitrary grazing angles ψ one obtains

$$\begin{aligned}
\mathscr{F} &= \frac{-2\tan^2\theta + 1}{2\tan^2\theta + 2\cot^2\psi + 1} \\
&= \frac{-2\frac{1-\cos(2\theta)}{1+\cos(2\theta)} + 1}{2\frac{1-\cos(2\theta)}{1+\cos(2\theta)} + 2\cot^2\psi + 1} \\
&= \frac{-2 + 2\cos(2\theta) + 1 + \cos(2\theta)}{2 - 2\cos(2\theta) + 1 + 2\cot^2\psi + (1 + 2\cot^2\psi)\cdot\cos(2\theta)} \\
&= \frac{-1 + 3\cos(2\theta)}{3 + 2\cot^2\psi + (2\cot^2\psi - 1)\cos(2\theta)}.
\end{aligned} \tag{9}$$

The relation between θ and figure of merit $\mathscr{F}$ is then:

$$\begin{aligned}
3\mathscr{F} + 2\cot^2\psi\cdot\mathscr{F} + (2\cot^2\psi - 1)\cdot\mathscr{F}\cdot\cos(2\theta) &= -1 + 3\cos(2\theta) \\
\cos(2\theta) &= \frac{-1 - 3\mathscr{F} - 2\cot^2\psi\cdot\mathscr{F}}{(2\cot^2\psi - 1)\cdot\mathscr{F} - 3}.
\end{aligned} \tag{10}$$

And finally,

$$\theta = \frac{1}{2}\arccos\left[\frac{1 + (2\cot^2\psi + 3)\cdot\mathscr{F}}{3 - (2\cot^2\psi - 1)\cdot\mathscr{F}}\right]. \tag{11}$$

2.3 Validation of the model: linearly polarized nitrogen K-edge spectra

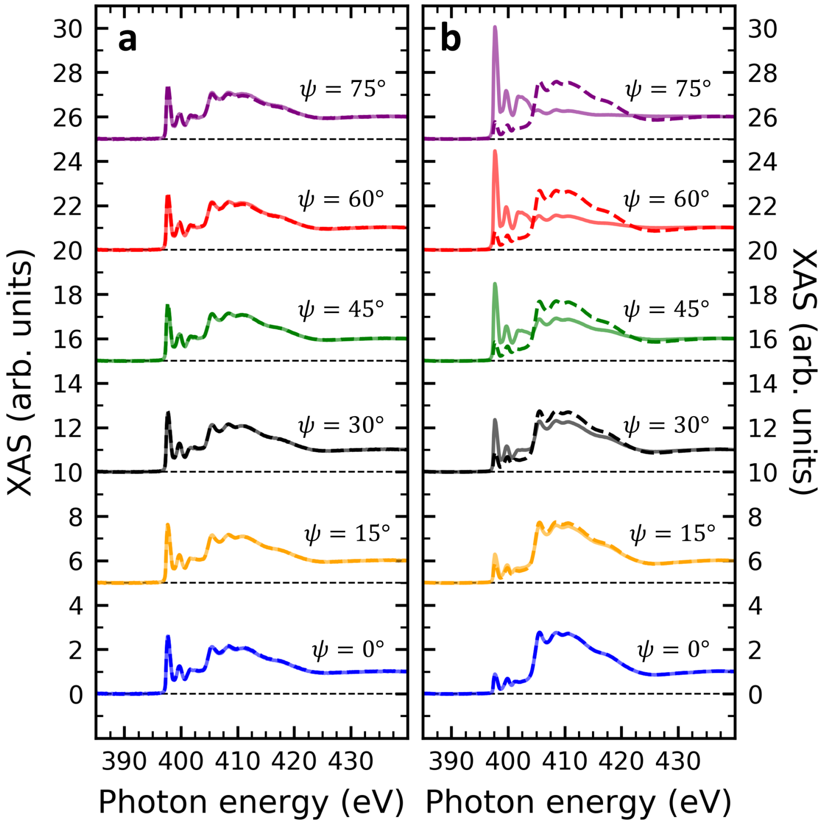

Figure S5. Nitrogen K-edge XAS from which the XLD shown in Figure 4 of the main text were extracted. The spectra were recorded on samples (a) `V10-Si` and (b) `V10-P-Si` at different grazing angles from $\psi = 0^\circ \ldots 75^\circ$ at intervals of 15°. Solid and dashed lines represent linear horizontal and vertical polarization of the X-rays, respectively.

3 Further experimental results

3.1 Additional XMCD spectra

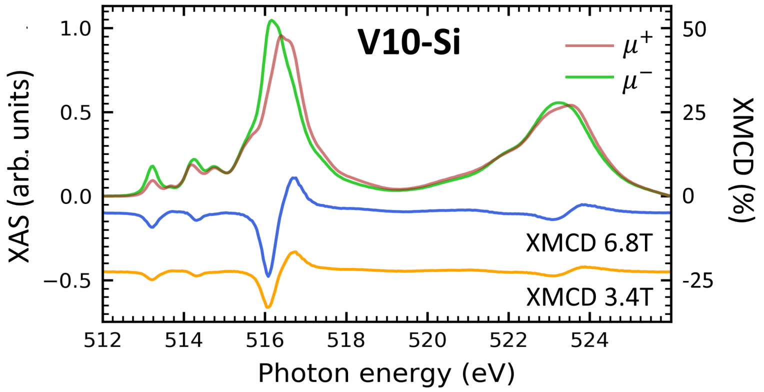

Figure S6. Circularly polarized XAS and XMCD at the vanadium $L_{2,3}$-edges of sample `V10-Si`. The XMCD curves are vertically offset for clarity. The spectra were obtained at 2 K at grazing incidence $\psi = 60°$ and at an applied magnetic field of 6.8 T unless stated otherwise. The XMCD scale is relative to the maximum feature of the XAS.

3.2 Results of the profilometer study

A Dektak 8 stylus profilometer was used to measure the thickness of the molecular films on sample `VT500-P-Si` and on dedicated calibration samples in order to verify the accuracy of the sublimation rates estimated by the in-situ quartz crystal microbalance. The scanning force was set corresponding to a mass of 0.1 mg. Step edges were deliberately created by masking certain areas of the Si substrates by narrow stripes of Kapton tape.

In Fig. S7 a representative profilometer scan is plotted clearly showing the step edges of the molecular film. A number of spikes and irregular steps are observed originating from the displacement of molecules by the tip. In order to improve the data quality, about 10 step edges were measured per calibration sample. Specifically, 15 step edges were sampled on the diluted VOPc film `VT500-P-Si`. The statistics on the step edges yielded an average value of 490 nm and a standard deviation of 80 nm consistent with the estimation of the quartz crystal microbalance using a conversion factor of $\Delta f/\Delta t = 1$ Hz/nm. This demonstrates that the microbalance is suitable to obtain a valid thickness estimation.

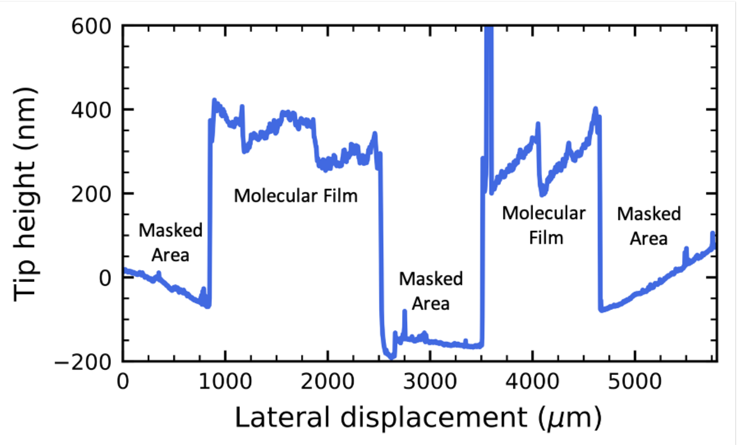

Figure S7. Representative profilometer scan obtained on sample `VT500-P-Si` on which a total frequency shift of 5 kHz was measured by the quartz crystal microbalance.
